# Physiological and Behavioral Responses of Stabled Horses (*Equus caballus*) to Three Types of Environmental Enrichment

**DOI:** 10.3390/ani15192779

**Published:** 2025-09-23

**Authors:** Miranda Brauns, Ahmed Ali, Jeannine Berger, Amy McLean

**Affiliations:** 1 Animal Biology Department, University of California Davis, Davis, CA 95616, USA; 2 Animal and Veterinary Sciences Department, Clemson University, Clemson, SC 29634, USA; ali9@clemson.edu; 3 Sacramento Veterinary Behavior Services, Vacaville, CA 95688, USA; drbergermvp@gmail.com; 4 Animal Science Department, University of California Davis, Davis, CA 95616, USA

**Keywords:** equine, welfare, behavior, physiology, enrichment, foraging, locomotion, frustration behavior, heart rate, smart halter

## Abstract

Small stalls and regulated feedings restrict horses’ natural foraging and locomotion, increasing risks to welfare. Environmental enrichment may promote more naturalistic behavioral time budgets, yet little is known about how enrichment type or timing affects physiology and behavior. This study examined nine stabled Quarter Horses provided with hay feeders, activity balls, or mirrors across randomized trials. Each trial included 30 min observations, four times per day, with enrichment removed between sessions and 5-day washouts between trials. Nightwatch^®^ Smart Halters™ recorded heart and respiration rates, while behaviors were video-scored using instantaneous scan sampling. Observers were not blind to the treatments. Enrichment effects, item type, time of day, and possible interactions for each variable were tested using a GLMM; Tukey’s HSD multiple comparison procedure was used for post hoc comparisons (at *p* ≤ 0.05). Enrichment significantly increased heart rate compared with the control (*p* = 0.03), indicating heightened arousal, with hay feeders producing the strongest effects. Respiration rate was unaffected. Mirrors reduced evening heart rates compared with other times (*p* = 0.02). Across treatments, enrichment increased foraging (*p* = 0.01) and locomotion (*p* = 0.03), while reducing frustration behaviors (*p* = 0.03). Hay feeders produced time budgets most similar to wild horses, suggesting greater effectiveness at meeting behavioral needs. Effects were most pronounced at 12:00 h and 16:00 h, outside routine meals. Overall, enrichment may improve physiological and behavioral outcomes, supporting its role in promoting welfare for stabled horses. Larger studies are needed to assess item-specific and long-term impacts.

## Simple Summary

Free-roaming horses travel around twenty miles per day and spend 50–60% of their time grazing, but domesticated horses often have limited time in turnout pastures and spend most of their day in stalls. This increases time standing stationary and greatly reduces grazing time, which can negatively impact the horses’ physical and mental health. Enrichment can help improve welfare by allowing the opportunity to perform species-typical behaviors and engage in mental and physical stimulation; therefore, this study compared how different enrichment items (hay feeders, activity balls, and mirrors) affected horse behavior and physiology. Enrichment did not affect respiration rate; however, it increased heart rate, grazing, and locomotor movement and decreased frustration behaviors. Effects were stronger during midday when horses did not have routine meal provisions. In our study, the hay feeder had stronger effects, though all items were effective enrichment and could be provided to improve the behavior, health, and welfare of stabled horses.

## 1. Introduction

Welfare is measured by an individual’s physiological, psychological, and behavioral state within their living conditions [1,2,3]. Enhancing welfare requires meeting both physical and mental needs in line with the species’ biology and natural history [1,2,4]. For horses, the ability to perform species-typical behavior is imperative for maintaining body condition, health, and positive mental states [5]. Ideally, horses are housed in large grassy paddocks (150–450 m^2^) [6,7,8,9], but management or land limitations often result in smaller, barren paddocks (10 m × 30 m [10]) or stalls (≈3.66 m^2^ [12 ft^2^]) [9,11,12,13,14]. While stalls provide accessibility, weather protection, and controlled feedings [15], limited turnout [7] and stall size restrict natural expression of behaviors. Studies show that welfare problems arise when an animal cannot carry out motivated behaviors [2,5,16].

Compared with free-roaming horses that graze for about 50–60% of the day and stand for 20–35% of it [17,18], stabled horses spend more time standing (40–58%) and less time eating (36–47% [15,19]). This discrepancy can impair musculoskeletal health [20] and digestion, increasing risk of ulcer development from continuous gastric acid secretion [21,22,23,24,25]. Reduced behavioral expression may also lead to negative subjective feelings such as increased stress [2,5,26] and frustration or stereotypical behaviors [26,27,28].

Enrichment seeks to mitigate these risks by providing stimulation that promotes positive experiences, provides opportunity and choice to perform species-typical behavior, and/or mitigates unwanted behaviors that may harm the animal’s well-being [5,29,30]. Successful enrichment improves biological functioning, cognition, and/or positive emotional states [5,29,31]. Evaluating enrichment is commonly realized via behavioral observations [32,33], although inactive use of enrichment may still provide welfare value due to enrichment offering choice, fulfilling behavioral motivations quickly, and signaling favorable environments [34]. Physiological measures, such as heart rate, can be directly measured to evaluate health [29] and could help identify the emotional affective state of the animal. Affective states are differentiated based on arousal (i.e., intensity of physiological activation; high or low) and valence (positive or negative) [35]. While physiology can reveal arousal levels [36], alone, they cannot distinguish valence [37]. Combining physiological and behavioral data could give insight into whether enrichment elicits positive or negative responses [38]. However, only 11% in a review of enrichment studies reported inclusion of physiological measures [32]. Therefore, although it is intuitively appealing to assume that enrichment will improve animal welfare, our understanding of how and to what extent enrichment influences horse behavior and physiology is limited.

Research on equine enrichment has largely focused on foraging devices (e.g., slow feeders and hay nets), as well as spatially and/or temporally distributed food provisions [39,40,41,42,43,44,45,46,47]. This type of enrichment has been reported to increase feeding time, reduce standing [39,40,45,46,47], and reduce frustration or stereotypical behaviors [41,42,46], though results vary [44,45]. Mirrors aim to alleviate stress associated with social isolation [48,49,50] and reduce stereotypies [48,49,50], although effects on normal behaviors are inconsistent [48,49]. Toys (e.g., Horseman’s Pride Jolly balls) encourage exploration, locomotion, and play, but often show limited engagement and minimal behavioral impact [43,44,51].

While it is difficult to compare enrichment due to differences in study designs, few studies have directly compared multiple enrichment types in a single project [43,44,51,52,53], leaving gaps in understanding relative effectiveness. Physiological measures associated with enrichment exposure are particularly underexplored in horses. While some evidence suggests music reduces stress indicators [54,55,56,57], little is known about how physical enrichments influence physiology alongside behavior.

With the objective to better understand enrichment effects so we can more effectively utilize enrichment to improve horse health and welfare, this study investigated the physiological and behavioral impacts of three common enrichments of differing sensory stimuli—hay feeders (gustation), activity balls (tactile), and mirrors (visual)—on stabled Quarter Horses. We examined both heart rate and respiration rate, as well as possible time-of-day effects to provide a comprehensive evaluation. We hypothesized that providing enrichment would increase heart and respiration rates relative to the control, increase foraging, locomotion, and enrichment-directed behaviors, and decrease frustration behaviors. We further predicted that the hay feeder would show the strongest effects among enrichment treatments and that enrichment impacts would be greater at 12:00 h and 16:00 h compared with 08:00 h and 20:00 h.

## 2. Materials and Methods

### 2.1. Animals and Housing

Nine quarter horses, comprising six geldings and three mares, with an average age of 11.33 ± 4.18 (mean ± SD) years and a 5.5 body condition score (1–9 scale [58]) were included in this study. All horses were individually housed in stalls with an attached dirt paddock and bedded with pine shavings. The horses were considered healthy and active members of the University of California Davis’s Division 1 Women’s Equestrian Team as part of the National Collegiate Equestrian Association (NCEA).

They were housed at the University of California Davis’s Equestrian Center in an open-air, center aisle style barn. Stalls measured 3.66 m. × 7.32 m. (12 ft. × 24 ft.), divided into two equal sections: (1) a 3.66 m^2^ (12 ft^2^) covered area with solid metal half walls (1.2192 m; 4 ft high) with bars on top (an additional 0.6096 m.; 2 ft. high), and (2) a 3.66 m^2^ (12 ft^2^) uncovered paddock area with metal bar half walls (1.397 m.; 4 ft. 7 in. high). All testing was performed at the Equestrian Center in each horse’s individual stall. Horses remained in their stall assigned via the Equestrian Center, so six study horses had two adjacent neighbors, and three study horses housed in the end-cap stalls had one adjacent neighbor, with visual and tactile contact between conspecifics available through the stall bars.

Horses remained in their stalls during the full duration of each research observation, and normal day-to-day husbandry management remained unchanged from their non-study routine, which included daily time outside of their stall for at least one and a half hours of training and 30 min of turnout. Stalls were cleaned of waste daily, and new bedding (a minimum of 2 bags per stall) was added to the covered stall areas weekly and as needed. Horses were provided three meals per day, the amount of which was calculated for individual horses at 1.5–2% of body weight, including (1) grass hay in the morning (07:30–08:30 h), (2) a commercial grain diet (LMF Stable Mix or LMF Senior concentrate pellets, Elk Grove Milling Company, Elk Grove, CA, USA) in the afternoon (16:00–19:00 h), and (3) alfalfa hay in the evening (18:30–19:30 h). Morning and evening hay provisions were provided on the ground. In addition to the normal provisions, one horse was given a lunch feeding (12:00 h) via a hay net hanging at about 0.46 m. (1 ft. 6 in.) off the ground. Concentrates were provided in a feed bucket at about 1.03 m. (3 ft. 4 in.) high off the ground. An automatic drinking water bowl was available in each stall, attached to the wall at a height of about 1.03 m. (3 ft. 4 in.), and water was accessible at all times.

This research was conducted in April 2022, with an average temperature of 15.5 °C (59.9 °F) and a humidity of 42.6% [59]. All research days were sunny and partly cloudy. This study was approved by the University of California Davis’s Institutional Animal Care and Use Committee (protocol #22748).

### 2.2. Experimental Design

The horses were monitored over three trials, with each trial consisting of 2 days: a control day with no enrichment item and an enrichment day with one of the three tested items: a hay feeder, an activity ball, or a mirror. Control days were always tested on day 1, and enrichment days were tested on day 2 for each trial. To minimize order effects of enrichment exposure, the nine horses were randomly divided into three groups of three, with each group receiving a different enrichment item per trial and rotating through the three enrichment items in subsequent trials: group A received the hay feeder (trial 1), mirror (trial 2), and activity ball (trial 3); group B received the mirror (trial 1), activity ball (trial 2), and hay feeder (trial 3); group C received the activity ball (trial 1), hay feeder (trial 2), and mirror (trial 3). By comparing data of an individual with and without enrichment, each horse served as its own control. The brand, color, and size of the enrichment items are outlined in Table 1. Grass hay, separate from the daily meal provisions, was used to fill the hay feeders, which were refilled to full capacity for each observation.

The horses were monitored for 30 min at four evenly spaced time points per day (08:00 h, 12:00 h, 16:00 h, and 20:00 h). The horses were equipped with a smart halter (Nightwatch^®^ Smart Halter™, Protequus, Austin, TX, USA [60]) 15 min before each time point to allow for desensitization of the halter before each observation started. On enrichment days, the enrichment item was introduced at the beginning of the hour at each observation start, with the hay feeder and activity ball being placed in the center of the covered area of the stall and the mirror being hung on the metal bar of the front right wall of the stall with hooks and secured with tying rope. Both the Smart Halters™ and enrichment items were removed between each observation. Following the enrichment day, a 5-day (120 h) washout period was held between each trial. This study continued until all three enrichment items were tested for all three groups, spanning a total of three weeks.

Observations were recorded via video capture (Lorex 4K Ultra HD NVR and Security Cameras, Lorex Corporation, Columbia, MD, USA), from which behavior was later scored. Cameras were set up on the ceiling of the center aisle of the barn, at about 3.51 m. (11 ft. 6 in.) off the ground and pointed toward the stall. This setup allowed for full visibility of the entire stall, except for a small angle directly behind the solid half walls in the front of the stall and separating the covered and uncovered stall sections, putting the horse “out of view” only if its head was completely lowered in that area. Each horse was monitored by an individual camera. During the observation sessions on both control and enrichment days, the research team stood at the entrance of the barn, away from individual stalls, so as not to influence behavior from their constant presence. Every 5–10 min, one to two researchers walked through the barn to visually check that all halters were still on and functioning (denoted by a small light on the left side of the halters behind the ear). The video capture was monitored during observations to ensure halter and horse safety, i.e., to stop horses from excessively biting/pulling the halters of neighboring horses, or if a horse showed adverse reactions to an enrichment item, the team could then remove the item for the rest of the session. Since the horses were already desensitized to people walking and talking through the barn as part of normal barn operations, and behavior was recorded in intervals (Section 2.3), the occasional moments of researchers walking through the barn were assumed to have minimal or no observer effect on the data.

### 2.3. Dependent Variables

Recorded videos of the observations were scored using the Behavioral Observation Research Interactive Software (BORIS; version 7.13.6 [61]). Behavioral data were recorded using focal animal, instantaneous scan sampling with 30 s intervals equating to 61 scans per observation per horse. Animal sampling was conducted by three observers, each being delegated one of the three trials for all nine horses. The behaviors recorded and operational definitions are listed in the ethogram (Table 2).

Physiological data were auto-recorded around every 21 s from the Nightwatch^®^ Smart Halters™. Outputs were given as beats per minute (heart rate) and breaths per minute (respiration rate) based on the average of the previous three successive readings using a moving average low-pass filter. Eighty-six data points were collected per observation for heart rate and respiration rate.

### 2.4. Statistical Analysis

To determine an appropriate sample size, a power analysis based on the study parameters (the number of dependent variables and observations) was conducted by Dr. Francisco Javier Navas González, the Editor-in-Chief of *Zootechnics Archives* and an academic researcher at the University of Córdoba (Spain). Since our study involved a 1:2 ratio of parameters to observations per study individual, the Bayesian approach was used to determine sample size because it is more appropriate for evaluating smaller datasets while retaining power and precision [63,64]. With an alpha value of 0.01, calculations indicated a minimum of nine animals per treatment group was needed.

Statistical analyses were performed by Dr. Ahmed Ali, Assistant Professor at Clemson University, using the R “stats” package software (version 3.3.1 [65]). The psych package was used to calculate descriptive statistics, which were presented as a mean ± standard error of the mean (SEM). Statistical significance was set at *p* ≤ 0.05. The block study design of the trial setup, in which there was a separate control day for each enrichment day, and each enrichment day testing all items divided amongst the horses, minimized possible day effects by allowing for an analysis of three control days versus three enrichment days. The effects of enrichment type, time of day, and all possible interactions on each variable (i.e., across treatments within times and within treatments across times) were evaluated using generalized linear mixed models (GLMMs) with the lme4 package [66]. Heart rate and respiration rate were first averaged for each 30 min observation and then used in the GLMM with the family set to “Poisson.” Behaviors were calculated as percentages (the number of occurrences of a specific behavior divided by the total number of observations at each time point multiplied by 100) and then used in the GLMM with the family set to “binomial.” Individual horses were entered as a random effect for all models. The residual distribution and assumptions for the GLMM of behavioral data were tested with the “DHARMa” package; the normality of the model residuals for physiological data was evaluated with the Shapiro–Wilk test. Tukey’s honestly significant difference (HSD) multiple comparison procedure was conducted using the multcomp package [67] for post hoc analyses of statistically significant effects of all models.

Inter-observer reliability was calculated during a 2 h animal sampling training when the observers performed behavior video decoding on the same four 15 min videos that were not included in the current study. As the instructor, the first author’s (M.B.) behavioral scoring of the same four videos was used as a comparison for the agreement measure when calculating reliability. Cohen’s kappa agreement coefficient (κ) was used [68], and inter-observer agreement was considered good when kappa exceeded 0.90. Observer reliability was reassessed at three evenly spaced time points throughout the data collection period to ensure reliability was maintained.

This study included 216 observations totaling 108 h of video (12 h per horse). One horse’s physiological data was excluded from this study due to technological problems with its smart halter. Excluded behavioral data included 0.08% due to horses being “out of view” and one observation for one horse due to the horse having a non-movable vet visit during the research session.

## 3. Results

### 3.1. Physiology

#### 3.1.1. Heart Rate

The interaction between time of day and treatments for heart rate was significant (*p* = 0.034). Among the individual treatments, enrichment significantly increased heart rate compared with the control at all time points, and there were some significant differences between the enrichment treatments (Table 3). Within the mirror treatment, horses had a lower heart rate at 20:00 h compared with 08:00 h, 12:00 h, and 16:00 h (*p* = 0.026, *p* = 0.031, and *p* = 0.029, respectively; Table 3). There were no differences in heart rate within the other treatments across times of the day.

#### 3.1.2. Respiration Rate

The interaction between time of day and treatments for respiration rate was not significant, so there were no differences across treatments or across times of the day.

### 3.2. Behavior

#### 3.2.1. Enrichment Interaction

The interaction between time of day and treatments for enrichment interaction was significant (*p* = 0.036). Within the mirror treatment, the enrichment interaction was higher at 12:00 h (19.52 ± 4.52; percentage of observation) compared with 08:00 h (7.85 ± 1.36; percentage of observation; *p* = 0.029) and 20:00 h (5.22 ± 1.03; percentage of observation; *p* = 0.036). The interaction was also higher at 16:00 h (18.52 ± 3.63; percentage of observation) compared with 08:00 h (*p* = 0.036) and 20:00 h (*p* = 0.038). There were no differences between 12:00 h and 16:00 h, nor between 08:00 h and 20:00 h. There were no differences within the hay feeder or activity ball treatments across times of the day, nor between the three enrichment treatments.

#### 3.2.2. Foraging

The interaction between time of day and treatments for foraging was significant (*p* = 0.032). Among the individual treatments, enrichment significantly increased foraging compared with the control at 08:00 h, 12:00 h, and 16:00 h, and there were some significant differences between the enrichment treatments at 1200 h (Table 4). Within the control, foraging was higher at 08:00 h compared with 12:00 h and 16:00 h (*p* = 0.021 and 0.027, respectively) and was also higher at 20:00 h compared with 12:00 h and 16:00 h (*p* = 0.023 and 0.026, respectively); however, there were no differences between 08:00 h and 20:00 h nor between 12:00 h and 16:00 h (Table 4). There were no differences within the enrichment treatments across times of the day.

#### 3.2.3. Locomotion

The interaction between time of day and treatments for locomotion was significant (*p* = 0.036). Among the individual treatments, the hay feeder and activity ball significantly increased locomotion compared with the mirror treatment and control at 08:00 h, 12:00 h, and 16:00 h (Table 5). Within the hay feeder treatment, locomotion was higher at 12:00 h compared with 08:00 h (*p* = 0.039) and 20:00 h (*p* = 0.035), and was higher at 16:00 h compared with 08:00 h (*p* = 0.034) and 20:00 h (*p* = 0.029). Similarly, within the activity ball treatment, locomotion was higher at 12:00 h compared with 08:00 h (*p* = 0.038) and 20:00 h (*p* = 0.026), and was higher at 16:00 h compared with 08:00 h (*p* = 0.031) and 20:00 h (*p* = 0.036). However, there were no differences between 12:00 h and 16:00 h, nor between 08:00 h and 20:00 h (Table 5). There were no differences within the mirror treatment or the control across times of the day.

#### 3.2.4. Frustration Behavior

The interaction between time of day and treatments for frustration behavior was significant (*p* = 0.013). Among the individual treatments, enrichment significantly decreased frustration behavior compared with the control at 12:00 h and 16:00 h, and there were no differences between the enrichment treatments (Table 6). There were no differences within the control or any of the enrichment treatments across times of the day.

#### 3.2.5. Other Behaviors

This project also analyzed the following behaviors: standing alert, standing rest, lying down, defecation, social behavior, and other (e.g., drinking, urination, grooming, etc.). Since standing behavior is inversely correlated with locomotion and significant differences were limited in the other behaviors, we chose to highlight only the key findings of foraging, locomotion, and frustration for the purpose of this paper. For comprehensive time budgets including the additional behaviors, please see Appendix A.

## 4. Discussion

### 4.1. Engagement with Enrichment

There were no significant differences in interaction behavior, so all three items appeared equally engaging to the horses. In contrast, Jørgensen et al. [43] found that horses in individual paddocks performed more item-directed behaviors toward edible items than non-edible items. Bulens et al. [51] found that while Jolly balls appeared as equally interesting as ropes, both had limited item-related behavior and had no effect on normal or abnormal behaviors of stabled horses. They concluded that non-edible items are not useful enrichment items for appropriately reared horses. In our study, all items had similar interaction times and significant differences in heart rate and behavior from the control. Thus, we argue that non-edible items such as activity balls and mirrors should be considered effective enrichment.

### 4.2. Physiological and Behavioral Effects

All enrichment items significantly increased heart rate compared with the control. This could be due to increased locomotion, as seen with the hay feeder and activity ball. However, heart rate was also elevated at 20:00 h and with the mirror, despite no differences in locomotion. Additionally, when heart rate is increased due to activity, an associated increase in respiration rate is expected because the body needs more oxygen to handle the demand of working harder [69]. Supporting studies show that horses have locomotor–respiratory coupling at higher levels of activity, although coupling is not observed with slower activity speeds [70,71]. This could explain why the respiration rate results were not significant, despite increased locomotion, but supports the claim that activity is likely not the sole driver behind the increased heart rate. Rather, enrichment effects on heart rate could be due to the horse’s emotional affective state. Similarly, Safryghin et al. [72] found that horses with fewer behavioral responses to a pre-feeding experiment had higher heart rate and concluded that the heart rate was due to arousal rather than activity. Specifically, increased heart rate is associated with high arousal [37] and suggests that enrichment could be inducing excitement (positive valence) or anxiousness (negative valence). The reduced frustration behaviors compared with the control suggest that enrichment did not create stress, and welfare benefits were positive; however, valence cannot be adequately determined without cognitive bias tests, so additional research is needed.

Horses had a significantly higher heart rate when provided with the hay feeder, suggesting that horses were in a higher arousal state. This could be due to the hay feeder providing a food reward, as grazing is a strong behavioral need in horses. The fact that the horses appeared aroused to forage suggests that foraging needs were not fully satisfied in stall housing. This is because horses are adapted to eat high fiber, low energy foods throughout the day with only short durations (three to four hours) of feed intake interruptions [73], which contrasts with the larger, less frequent daily provisions. For example, horses on non-edible shavings eat regulated meals more quickly, resulting in a longer period (9 h overnight) of feed intake interruption [73]. Benhajali et al. [39] found similar results when horses given the opportunity to forage spent significantly more time feeding compared with horses in bare paddocks (65.12% vs. 29.75%). Likewise, horses significantly increased feeding time from hay bags (~40%) and slow feeders (~45%) compared with control hay on the ground (~28%) and reduced undesirable behaviors [45].

Many horses were observed to engage in contrafreeloading, i.e., horses started foraging from the hay feeder in which they had to work for food even if they still had provisions freely accessible on the ground. Others appeared to perform patch foraging, i.e., horses did not finish food at one source before switching to the other source, alternating between the hay feeder and daily provisions. Similarly, Winskill et al. [47] found that horses directed foraging toward a foodball instead of ad libitum hay, suggesting the foodball had a higher incentive value. Horses provided multiple sources of forage showed significantly more foraging and less alert behavior, locomotion, and eating of straw bedding compared with single-source forage [41]. Also, horses on a multiple-source diet increased foraging by about 20%, sampled all forages during observations, and did not perform stereotypical weaving as single-forage horses did [46]. Horses showing more species-typical foraging behavior when it was provided in alternate sources is supporting evidence for the welfare benefits of contrafreeloading and patch foraging [41,46,47]. While nutritional needs may be met, regulated feedings do not accommodate the horse’s motivation for natural foraging [73], while the hay feeder appeared to address such needs.

The increased foraging when provided with non-edible enrichment may be due to a novelty effect, encouraging horses to investigate the area and subsequently forage due to the environmental change of a new item in the stall. Conversely, McAfee et al. [49] found that mirrors did not affect ingestion behavior, and Bulens et al. [51] stated that Jolly balls seemed to have no effect on normal behaviors.

Regardless of item type, enrichment encouraged foraging to the extent that the behavior became more consistent throughout the day. Meanwhile, horses in the control group performed less foraging at 12:00 h and 16:00 h, resulting in inconsistent temporal foraging. While the effects of activity balls and mirrors on foraging’s temporal distribution are under-researched, Rochais et al. [45] similarly found that horses had a better temporal distribution of foraging when provided a slow feeder. They found that slow feeder horses always had hay available at midday, and 9.2% mean of horses had hay left near the end of the day, unlike those with the hay bag and the control group. Since consistent foraging is in line with horses’ natural adaptations [73], these results support improved welfare through the opportunity to fulfill behavioral needs, improve emotional states, and lower risk of empty gut fill and ulcer development.

Locomotion increased from the control when horses were provided with the hay feeder and activity ball. Standing stationary can impair musculoskeletal health [20], so increased locomotion supports improved welfare. Contrastingly, Benhajali et al. [39] found a decrease in locomotion when horses were given foraging opportunities compared with the control (11.70% vs. 23.56%). The difference could be the experimental area being a paddock rather than stalls. While the horses that received enrichment in both studies had similar percentages of locomotion, the control horses in Benhajali et al.’s study [39] had more locomotion (23.56% of scans) than the control horses in our study (2.56% of the observation), which influenced whether the behavioral change was an increase or a decrease. Winskill et al. [47] found that foodballs significantly decreased locomotion in stabled horses; however, they noted that this anomaly stemmed from their ethogram incorporating moving and eating during enrichment interaction as one category. Thus, the increased locomotion from the current study is likely due to the items needing to be moved for foraging, with the hay feeder needing to be manipulated to access food and the activity ball needing to be moved away from provisions on the ground. In addition, the activity ball created a larger obstacle in the stall that horses need to walk around when locomoting. It could be argued that this reduces the already limited space; however, horses provided the activity ball showed species-typical time budgets (Appendix A) and performed fewer frustration behaviors than the control at 12:00 h and 16:00 h, suggesting that the activity ball did not add stress.

Frustration is often exhibited by horses and other animals when in a compromised or stressful environment [38,74]. They can even become repetitive and may contribute to the development of stereotypies [26,27,28,74]. Therefore, the reduced frustration behaviors seen in all enrichment treatments indicate improved welfare associated with better fulfillment of behavioral needs, lowered stress and/or boredom, and positive emotional states. It also suggests that enrichment could help mitigate stereotypical behaviors from developing. This was not analyzed in the current study, but other studies show that certain enrichments do reduce stereotypies in horses [42,48,49,50,52]. Henderson and Waran [42] similarly concluded that enrichment may help create an environment less likely to lead to the development of abnormal behaviors when used in conjunction with other measures. Further research utilizing a sample size of horses with stereotypies is needed to better understand enrichment effects on these behaviors.

### 4.3. Time-of-Day Effects

No significant differences in behavior were found between the control and enrichment treatments at 20:00 h. Additionally, the mirror had less enrichment interaction and a smaller effect on heart rate at 20:00 h compared with other times. The limited reflection that could be seen in the darker light conditions may have reduced the mirror’s physiological and behavioral effects. Similarly, Mills and Davenport [50] found that horses provided a mirror performed more alert and stereotypic behaviors during the evening compared with the morning and midday. This suggests that enrichment—particularly mirrors—should likely be implemented during daylight hours for more effective welfare benefits.

Conversely, Jørgensen et al. [75] found that interest toward a concentrate ball and toy ball was lowest in the afternoon. Bulens et al. [52] found that horses displayed more item-related behaviors toward sand-filled bottles and rope in the evening, but also that horses showed a higher frequency of item-related behavior when no hay was available. Our results of the hay feeder and activity ball showing no difference in interaction across times could be due to both items needing to be pushed for foraging during daily provisions at 08:00 h and 20:00 h, while also being engaging at 12:00 h and 16:00 h when the stall was otherwise barren. Despite being engaging at all times, a greater number of behaviors were significant at 12:00 h and 16:00 h when horses did not have daily provisions, suggesting a time-of-day effect. Within the hay feeder and activity ball treatments, locomotion was higher at these times, and frustration behaviors were only significantly different from the control at these times, along with several other behaviors analyzed in the full study (i.e., standing alert, social, and other behaviors; Appendix A). Winskill et al. [47] also found that time of day influenced enrichment effects for all behaviors except elimination when testing the effects of a foodball; however, they found peak durations of foodball interaction at sunrise and sunset. The contrasting results are likely due to their study observing horses overnight as opposed to during the day, but support our conclusion that enrichment seems to have more effective benefits when horses are not otherwise preoccupied fulfilling higher incentive behavioral needs (e.g., daily meal provisions and sleep).

### 4.4. Overall Evaluation

As previously defined, enrichment should only be evaluated as successful if it provides an environment that promotes positive experiences and species-typical behaviors [5,76]. Leiner and Fendt [38] concluded that physiological and behavioral fear responses in horses are positively correlated, meaning that an increased heart rate associated with a negative experience would likely result in stress-related behaviors (e.g., flattened back ears, eye whites, tail swishing, snorting, trembling, and sweating) and/or avoidance behaviors (e.g., leaning or stepping away, and startle/flight response). The study results, in which horses that showed stress-related behaviors in novel object tests had a significantly higher heart rate increase than horses that did not show stress behaviors [72], support this claim. We noticed slight startle behavior when the mirror was first introduced, but did not notice any other stress or avoidance responses during enrichment treatments (personal observation). The results supporting the lack of behavioral fear include horses spending 13.36% of observations engaged with any of the three enrichments, and frustration behavior significantly decreased compared with the control. Furthermore, state anxiety (high arousal and low valence) is often accompanied by an increase in respiration rate [77], but our results found no difference in respiration.

Limited research has analyzed the physiological effects of enrichment on horses; however, music has been recorded to lower heart rate, heart rate variability, and cortisol [54,55], reduce stress responses when exposed to transport and farriery [56], and have positive effects on emotional states [78]. While music appears to induce low arousal rather than high arousal, as shown in the current study, the positive behavioral responses suggest enrichment promotes affective states of positive valence. Further research involving cognitive bias testing, however, is needed to more accurately infer valence in response to enrichment. Overall, the physiological and behavioral results suggest the horses did not respond negatively and performed more species-typical behaviors, so we conclude that the hay feeder, activity ball, and mirror can be evaluated as effective enrichment.

There were limited differences between the three items, but the hay feeder had significantly higher foraging than the activity ball and mirror at 12:00 h, and the hay feeder and activity ball had significantly higher locomotion than the mirror at 08:00 h, 12:00 h, and 16:00 h. This indicates an enrichment-type effect for these behaviors. Overall, the hay feeder appeared to provide the most prominent changes in heart rate and behavior compared with the control and had a more species-typical time budget that was similarly consistent throughout the day (Appendix A). This suggests the hay feeder may be more effective as an enrichment item in fulfilling overall behavioral needs, but does not discredit the positive behavioral changes promoted by the activity ball and mirror. Facilities are recommended to use specific enrichment items to target behaviors based on their specific welfare goals for individual horse needs. Taylor et al. [31] also highlight this concept by concluding that focusing enrichment strategies on specific animal welfare outcomes, rather than the intent, will assist owners in identifying optimal enrichment for their purposes.

### 4.5. Other Considerations

Enrichment could encourage mental stimulation by adding complexity to behaviors and providing opportunities to investigate and problem solve. This is particularly true for mirrors, which provide a novel concept (reflection) that the horse may not have previously been exposed to or encountered very often, as mirrors are not present in the University of California Davis’s arena where the horses train. It is likely horses can perceive reflections due to the contrast of the images it is reflecting and horses’ eyes being extremely sensitive to movement [15]. McAfee et al. [49] concluded that mirrors may alter the horse’s perception of the environment by creating the illusion of visual contact with conspecifics and, thus, influence the resultant responses to that environment. Decker et al. [34] concluded that enrichment that elicits little to no interaction still has welfare benefits, such as reducing stress. So, while the hay feeder and activity ball had stronger behavioral effects, the mirror may be similarly effective as enrichment, given its possible mental stimulation. More research is needed to assess the cognitive effects of enrichment.

It is important to note that not all horses may respond the same way to an enrichment item. During this study, a horse was observed to pick up and shake the activity ball. This response may be personality-dependent. Reactions to enrichment may also be dependent on factors such as predisposition to perform stereotypic behaviors [53], age, and sex [52]. Additionally, some facilities may operate differently, which could alter results. Considering these points, managers should monitor when enrichment is initially introduced and experiment to see what items work best for their horse.

The limitations of the current study may have affected the results. Specifically, the sample size was small, and horses were only tested over one day for each enrichment item. Therefore, some results may have been due to novelty rather than the items themselves. Novelty can be an important component of effective enrichment, but it is equally important to uncover whether enrichment has positive effects when they are no longer novel. This was not analyzed in the current study, but several studies show that enrichment elicits effects over multiple days [43,46,49,52], which suggests that novelty may not be entirely necessary for enrichment to maintain its welfare benefits. However, due to this study’s limitations, more research is recommended to further investigate novelty and dissipating effects of repeated exposure to evaluate long-term benefits of enrichment.

In addition to research topics mentioned throughout the Discussion, other variables for investigation include additional types of enrichment items; enrichment effects based on type of housing, breed, age, personality, and/or other equine species; and a sample size of horses with stereotypical behaviors to further analyze enrichment type effects on those behaviors. Further research is recommended to analyze heart rate variability, cortisol, or cognitive bias testing to provide more insight into horses’ emotional valence toward enrichment. Most importantly, it is highly recommended that future studies utilize larger sample sizes and continuous sampling methods to obtain a closer representation of daily time budgets in order to fully understand enrichment effects on equine.

## 5. Conclusions

Enrichment did not affect respiration rate but did increase heart rate, which indicates that horses were in a high-arousal state when provided enrichment. The hay feeder showed the strongest physiological effects among the items, and heart rate was not dependent on provision time, except for horses with the mirror, having a higher heart rate during daylight hours.

Overall, enrichment encouraged more species-typical time budgets in stabled horses. All items had positive behavioral effects compared with the control and could be used as effective enrichment, though there were enrichment-type effects for certain behaviors, so enrichment program strategies could be used to target and achieve specific management goals. However, the hay feeder appeared more effective at fulfilling overall behavioral needs. Behavioral effects of enrichment were present at all time points except 20:00 h, but there were more differences from the control at 12:00 h and 16:00 h. Therefore, it is recommended to provide enrichment during times when horses do not have meal provisions.

In conclusion, this study gives evidence that providing enrichment has a positive effect on physiology and behavior and is strongly recommended to improve the behavioral needs, health, and overall welfare of stabled horses. However, it is important to consider that other variables (e.g., personalities and novelty) may shape how horses respond; hence, owners should evaluate what works best for their horse. More research is needed with larger sample sizes to study other items, affective states, and long-term effects.

## Figures and Tables

**Table 1:** Enrichment items used for the hay feeder, activity ball, and mirror treatments.

Item		Color	Size
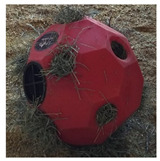	Hay feeder: High Country Plastics (Caldwell, ID, USA) Hay Play™ Slow-feeding forage ball	Red	16 in. diameter
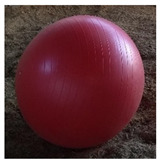	Activity ball: Horsemen’s Pride (Streetsboro, OH, USA) mega ball	Red	25 in. diameter
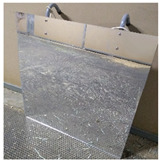	Mirror: Marketing Holders (Mims, FL, USA) acrylic mirror sheet	N/a	24 in. × 24 in.

**Table 2:** Ethogram of behaviors recorded during observations (adapted from [15,45,62]).

Behavior	Description
Foraging	The horse is searching for and/or grazing/chewing/eating food that did not directly come from the hay feeder enrichment item; the horse may be moving ^a^ around the stall with its head below the withers and its muzzle close to the ground or shifting through the substrate, and may or may not be actively chewing; or the horse may be standing stationary with its head either below or above the withers and is chewing food.
Locomotion	The horse has taken at least two footfalls or more forward or backward, at any pace, and is moving around the stall without interacting with the enrichment item.
Frustration behavior	The horse expresses frustration in one of the following ways: Stomping: The horse abruptly stomps foot on the ground, which may be repetitive. Kicking: The horse is kicking, which may or may not be directed toward a conspecific or person. Biting: The horse bites an object that is not food or the enrichment item, which may be repetitive. Pawing: The horse paws at the ground with its hoof, not followed by rolling. Repetitive behavior: The horse expresses repetitive behavior (more than three immediately consecutive instances of the same behavior) in the form of yawning, licking, or stereotypical behavior.
Enrichment interaction	The horse is interacting with the enrichment item in one of the following ways: Investigation: The horse is standing still and looking at the enrichment item; the horse sniffs the item with its muzzle close to or touching the enrichment item; and/or the horse startles (denoted by muscles tensing and jolt of the body and/or head with ears facing forward) and briskly retreats from the enrichment item. Push: The horse pushes the enrichment item with its muzzle, leg, or body. Rub: The horse rubs its head, leg, or body on the enrichment item. Lick: The horse uses its tongue to lick the enrichment item. Bite: The horse uses its lips to manipulate and teeth to bite on the enrichment item. Forage: The horse is searching for and/or grazing/chewing/eating food directly from the enrichment item ^b^.
Other	Any behavior not otherwise listed (e.g., drinking, urinating, grooming, and rolling).
Out of view	The horse’s head is lowered and completely hidden from view behind the stall wall and its behavior is unable to be accurately recorded.

^a^ If the horse was in locomotion while foraging, the behavior was scored as foraging if the horse’s head remained below the withers and was scored as locomotion if the horse’s head raised above the withers at the time of the scan. ^b^ As it was difficult to identify if hay on the ground came from the enrichment or daily provision, horses foraging from the ground was scored as regular foraging behavior, not enrichment interaction.

**Table 3:** Heart rate (mean beats per minute ± SE) of stabled horses (n = 8) when provided with the hay feeder, activity ball, mirror, and control during 30 min observations at different times of the day. *p*-values represent Tukey HSD post hoc multi-comparisons at *p* ≤ 0.05.

Time	Hay Feeder (HF)	Activity Ball (AB)	Mirror (MIR)	Control (CON)	*p*-Value (HF vs. AB)	*p*-Value (HF vs. MIR)	*p*-Value (HF vs. CON)	*p*-Value (AB vs. MIR)	*p*-Value (AB vs. CON)	*p*-Value (MIR vs. CON)
08:00 h	70.58 ± 3.52 ^A^	53.58 ± 3.22 ^B^	64.25 ± 5.06 ^Bx^	29.52 ± 3.88 ^C^	0.028	0.028	0.027	-	0.027	0.022
12:00 h	71.96 ± 4.25 ^A^	62.52 ± 4.25 ^A^	55.85 ± 6.52 ^Bx^	35.56 ± 4.58 ^C^	-	0.036	0.022	0.029	0.028	0.019
16:00 h	65.58 ± 6.52 ^A^	54.22 ± 4.20 ^B^	53.41 ± 4.58 ^Bx^	37.89 ± 4.02 ^C^	0.032	0.032	0.036	-	0.027	0.024
20:00 h	75.85 ± 3.25 ^A^	46.52 ± 3.85 ^B^	42.52 ± 5.52 ^By^	30.22 ± 3.99 ^C^	0.039	0.041	0.038	-	0.031	0.036

A–C superscripts represent statistically significant differences within the same time of day [row] and across different treatments [column]. x–y superscripts represent statistically significant differences within the same treatment [column] and across different times of the day [rows].

**Table 4:** Percentage of observation (mean ± SE) the stabled horses (n = 9) spent performing foraging behavior when provided with the hay feeder, activity ball, mirror, and control during 30 min observations at different times of the day. *p*-values represent Tukey HSD post hoc multi-comparisons at *p* ≤ 0.05.

Time	Hay Feeder (HF)	Activity Ball (AB)	Mirror (MIR)	Control (CON)	*p*-Value (HF vs. AB)	*p*-Value (HF vs. MIR)	*p*-Value (HF vs. CON)	*p*-Value (AB vs. MIR)	*p*-Value (AB vs. CON)	*p*-Value (MIR vs. CON)
08:00 h	60.23 ± 5.25 ^A^	53.85 ± 3.89 ^A^	55.63 ± 6.03 ^A^	49.85 ± 5.63 ^Bx^	-	-	0.035	-	0.038	0.041
12:00 h	57.99 ± 4.89 ^A^	44.52 ± 4.25 ^B^	45.66 ± 4.25 ^B^	30.56 ± 4.58 ^Cy^	0.036	0.029	0.029	-	0.036	0.032
16:00 h	54.36 ± 3.55 ^A^	45.23 ± 5.12 ^A^	47.96 ± 7.52 ^A^	31.58 ± 6.99 ^By^	-	-	0.025	-	0.023	0.039
20:00 h	59.85 ± 5.63	52.36 ± 6.63	53.59 ± 2.36	49.85 ± 6.87 ^x^	-	-	-	-	-	-

A–C superscripts represent statistically significant differences within the same time of day [row] and across different treatments [column]. x–y superscripts represent statistically significant differences within the same treatment [column] and across different times of the day [rows].

**Table 5:** Percentage of observation (mean ± SE) the stabled horses (n = 9) spent performing locomotion when provided with the hay feeder, activity ball, mirror, and control during 30 min observations at different times of the day. *p*-values represent Tukey HSD post hoc multi-comparisons at *p* ≤ 0.05.

Time	Hay Feeder (HF)	Activity Ball (AB)	Mirror (MIR)	Control (CON)	*p*-Value (HF vs. AB)	*p*-Value (HF vs. MIR)	*p*-Value (HF vs. CON)	*p*-Value (AB vs. MIR)	*p*-Value (AB vs. CON)	*p*-Value (MIR vs. CON)
08:00 h	7.23 ± 1.03 ^Ay^	8.69 ± 1.56 ^Ay^	4.25 ± 1.01 ^B^	3.63 ± 1.03 ^B^	-	0.039	0.031	0.043	0.019	-
12:00 h	14.03 ± 2.96 ^Ax^	16.25 ± 2.52 ^Ax^	3.69 ± 0.96 ^B^	1.66 ± 0.69 ^B^	-	0.026	0.042	0.023	0.031	-
16:00 h	15.99 ± 3.66 ^Ax^	14.52 ± 3.33 ^Ax^	4.03 ± 1.12 ^B^	1.25 ± 0.23 ^B^	-	0.032	0.025	0.037	0.036	-
20:00 h	5.66 ± 1.03 ^y^	7.58 ± 1.01 ^y^	9.85 ± 1.33	5.63 ± 1.03	-	-	-	-	-	-

A–B superscripts represent statistically significant differences within the same time of day [row] and across different treatments [column]. x–y superscripts represent statistically significant differences within the same treatment [column] and across different times of the day [rows].

**Table 6:** Percentage of observation (mean ± SE) the stabled horses (n = 9) spent performing frustration behaviors when provided with the hay feeder, activity ball, mirror, and control during 30 min observations at different times of the day. *p*-values represent Tukey HSD post hoc multi-comparisons at *p* ≤ 0.05.

Time	Hay Feeder (HF)	Activity Ball (AB)	Mirror (MIR)	Control (CON)	*p*-Value (HF vs. AB)	*p*-Value (HF vs. MIR)	*p*-Value (HF vs. CON)	*p*-Value (AB vs. MIR)	*p*-Value (AB vs. CON)	*p*-Value (MIR vs. CON)
08:00 h	2.66 ± 0.23	3.56 ± 0.22	4.36 ± 1.01	4.52 ± 1.12	-	-	-	-	-	-
12:00 h	0.66 ± 0.06 ^B^	1.03 ± 0.12 ^B^	2.99 ± 0.63 ^B^	7.89 ± 1.13 ^A^	-	-	0.035	-	0.041	0.027
16:00 h	0.89 ± 0.09 ^B^	1.12 ± 0.13 ^B^	2.03 ± 0.16 ^B^	11.96 ± 1.69 ^A^	-	-	0.031	-	0.041	0.043
20:00 h	2.01 ± 0.63	3.03 ± 0.96	2.69 ± 0.25	3.96 ± 0.98	-	-	-	-	-	-

A–B superscripts represent statistically significant differences within the same time of day [row] and across different treatments [column].

**Figure A1:**
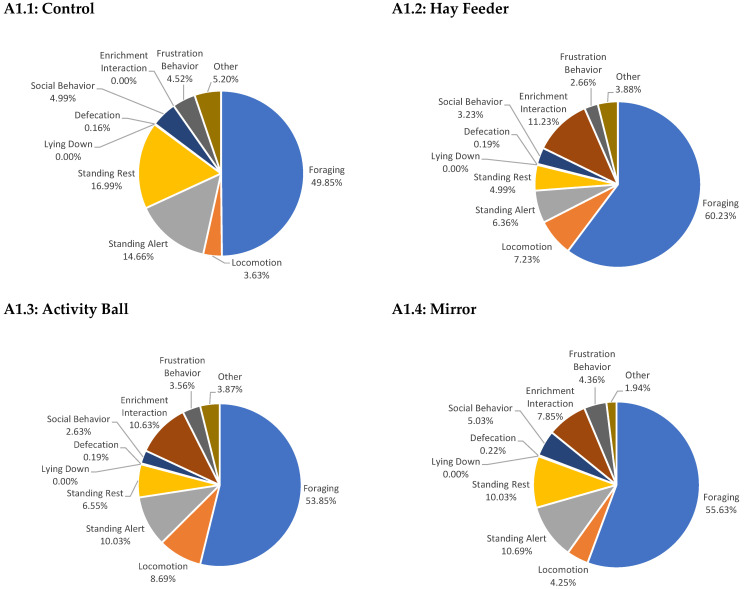
Time budgets of stabled horses at 08:00 h when provided with the hay feeder, activity ball, mirror, and control.

**Figure A2:**
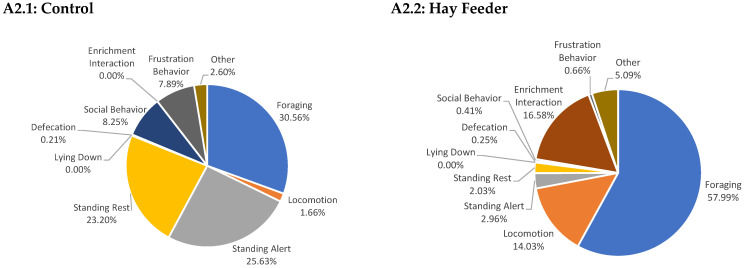

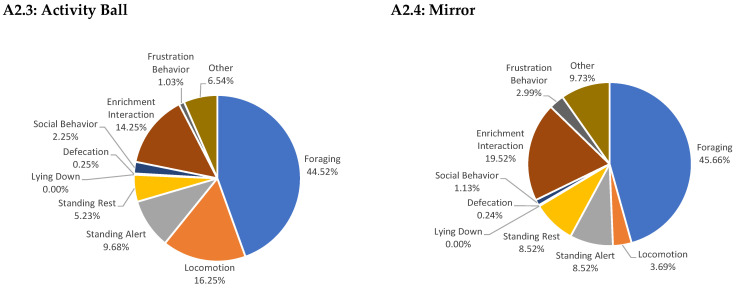
Time budgets of stabled horses at 12:00 h when provided with the hay feeder, activity ball, mirror, and control.

**Figure A3:**
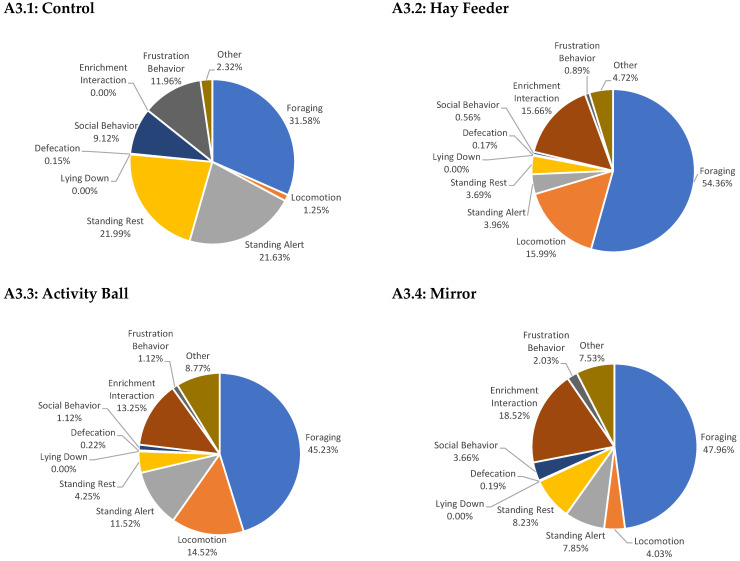
Time budgets of stabled horses at 16:00 h when provided with the hay feeder, activity ball, mirror, and control.

**Figure A4:**
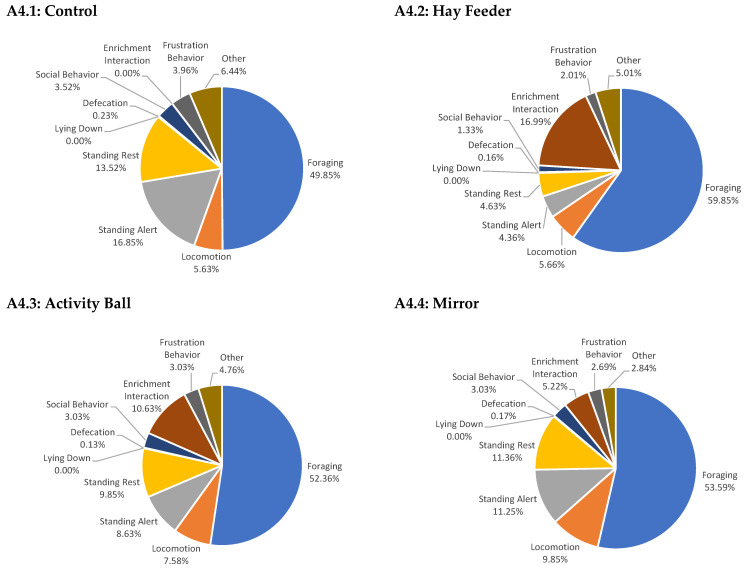
Time budgets of stabled horses at 20:00 h when provided with the hay feeder, activity ball, mirror, and control.

## Data Availability

The data will be made available from the corresponding author upon reasonable request.

## Abbreviations

The following abbreviations were used in this manuscript:

HF	Hay feeder
AB	Activity ball
MIR	Mirror
CON	Control

## Appendix A

This appendix includes the time budgets of the stabled horses in the control group and when provided with the hay feeder, activity ball, and mirror treatments at different times of the day. This offers a more comprehensive analysis of overall behavior, including the additional behaviors studied in the project but not overviewed in this manuscript (see Section 3.2.5). The following figures (Figure A1, Figure A2, Figure A3 and Figure A4) are made up of twelve subfigures. The values represent the mean percentages of observation in which the behavior was performed over 30 min observations across all animals with that treatment at the associated time of day. For additional information, please refer to the thesis manuscript available at {https://escholarship.org/uc/item/6wc24448} (accessed on 17 July 2025).

All three enrichment items had several significant differences compared with the control, with many of the effects being similar across enrichment items; however, there were some various effects as well. The hay feeder was the most influential among the three items at increasing foraging at 12:00 h. Unlike the other items, the mirror did not have a significant effect on locomotion at any time point. The hay feeder was more influential than the activity ball, but not the mirror, in reducing standing alert behavior at 16:00 h. Within each treatment, there was significantly more locomotion (within the hay feeder and activity ball treatments) and enrichment interaction (within the mirror treatment) at 12:00 h and 16:00 h compared with 08:00 h and 20:00 h, and there was more foraging (within the control group) at 08:00 h and 20:00 h compared with 12:00 h and 16:00 h.
